# A Successful Treatment of Broncho-Esophageal Fistula with Esophageal Stenting Using Direct Endoscopic Visualization

**DOI:** 10.3390/medicina60040524

**Published:** 2024-03-22

**Authors:** Christian Banciu, Adrian Aprotosoaie, Dorin Vancea, Sorina Taban, Cristina Guse, Oana Budu, Ramona Fabian, Sorin Chiriac, Florina Căruntu, Adrian Voicu

**Affiliations:** 1 Department of Internal Medicine IV, Faculty of Medicine, “Victor Babes” University of Medicine and Pharmacy, 2 Eftimie Murgu, 300041 Timisoara, Romania; banciu.christian@umft.ro (C.B.); adrian.aprotosoaie@gmail.com (A.A.); marin.cristina01@yahoo.com (C.G.); oanadaniela.budu@yahoo.ro (O.B.); rgerlinder@yahoo.com (R.F.); 2 Clinic of Pneumology I, Clinical Hospital of Infectious Diseases and Pneumophysiology Dr.Victor Babeș Timișoara, 300310 Timisoara, Romania; dovancea@yahoo.com; 3 Department of Histopathoogy, Faculty of Medicine, “Victor Babes” University of Medicine and Pharmacy, 2 Eftimie Murgu, 300041 Timisoara, Romania; taban.sorina@umft.ro; 4 Department of Surgery III, Faculty of Medicine, “Victor Babes” University of Medicine and Pharmacy, 2 Eftimie Murgu, 300041 Timisoara, Romania; chiriac.sorin@umft.ro; 5 Department Medical Semiology II, Faculty of Medicine, “Victor Babes” University of Medicine and Pharmacy, 2 Eftimie Murgu, 300041 Timisoara, Romania; 6 Department of Pharmacology—Pharmacotherapy, Faculty of Pharmacy, “Victor Babes” University of Medicine and Pharmacy, 2 Eftimie Murgu, 300041 Timisoara, Romania; adrian.voicu@umft.ro

**Keywords:** broncho-esophageal fistula, stenting, self-expanding metal stent (SEMS), lung carcinoma, bronchopulmonary adenocarcinoma

## Abstract

Broncho-esophageal fistula (BEF) is a severe yet relatively rare connection between the bronchus and esophagus usually caused by esophageal and pulmonary malignancies. We present a case report of a 49-year-old man diagnosed with terminal lung carcinoma who developed a BEF. The thoracic computed tomography scan detected a mass in the left bronchi that partially covers and disrupts the bronchial contour in certain regions and extends to the esophageal wall. After thoroughly evaluating alternative treatment approaches, we opt for the stenting procedure due to the advanced stage of the tumor and the significantly diminished quality of life. The treatment involves the use of a partially covered metal stent that is known to exhibit lower potential to migrate. The treatment is highly successful, resulting in a significant enhancement of the patient’s quality of life, a lengthening in his survival, and the ability to pursue additional palliative treatment options. In contrast to the typical prosthesis implantation, our procedure uses a direct endoscopic visualization for the proximal deployment of a partially covered stent, offering a cost-effective and radiation-free alternative that can be particularly beneficial for BEF patients in facilities without radiology services.

## 1. Introduction

**Figure 1:**
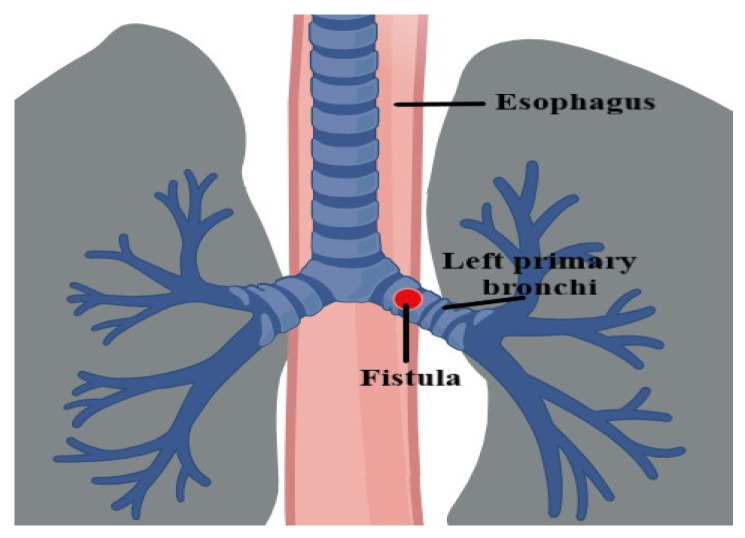
Broncho-esophageal Fistula (Created with BioRender.com).

Broncho-esophageal fistula (BEF) is an uncommon condition resulting from an abnormal connection between the bronchus and the esophagus that can be either congenital or acquired (Figure 1). In adults, congenital fistulas are quite rare; therefore, the occurrence of such cases usually suggests an acquired condition of benign (infections, chest traumatic events, prolonged endotracheal intubation) or malignant (esophagus, bronchi, mediastinum, lungs) origin [1,2]. Malignancy-associated broncho-esophageal fistulas may occur due to tumor infiltration and subsequent rupture, or as a result of radiation, laser therapy, chemotherapy, pre-existing stents (particularly esophageal stents), or a combination of these factors [3]; however, compared to tracheoesophageal fistulas, they are much rarer, with very few cases reported in the medical literature, which makes their diagnosis either delayed or inaccurate [4]. Additional causes of delayed diagnosis are the nonspecific and common symptoms such as cough, dysphagia, signs of aspiration, chest pain, or gastroesophageal reflux that may, however, severely impact the patient’s quality of life [5]. Most malignant broncho-esophageal fistulas develop as a complication of esophageal cancer, being present in 5–10% of affected patients; in lung cancers, this complication is much less frequently encountered and develops mainly for squamous cell carcinomas [4]. Although rare, the condition may lead to major morbidity and increased mortality rates in patients, being a life-threatening complication due to the risk of the development of recurrent pulmonary infections and sepsis [4,6]; the underlying cause should always be investigated. Treatment should be initiated promptly following the confirmation of the diagnosis to avoid sepsis and aspiration; the therapeutic approach must consider the symptoms, the configuration and location of the fistula, as well as its extent [7]. In the case of small lesions, chemotherapy alone or combined chemo/radiation therapy can be sufficient for complete resolution [4]. Other potential treatments involve surgery, stenting (either esophageal or bronchial), and endoscopic procedures (tissue adhesives, clips) [8]. However, the risk of infection or bleeding is very high, and most patients suffer fatal complications within 3 months after intervention [8].

## 2. Case Presentation Section

A 49-year-old man, heavy smoker with a history of chronic alcohol consumption, presented with general physical weakness and lack of energy, weight loss, and lack of appetite, symptoms that started four months ago. In the last two weeks, his condition worsened with high-grade dysphagia and a persistent mucopurulent cough with foul-smelling sputum. The physical examination of the lungs revealed bronchial and crackle rales in the left lung area.

The results of the biological test were within the usual range (Table 1). His sputum grew 50% *Neisseria* spp., 40% St. viridans, and 10% negative gram bacilli.

### 2.1. Thoracic Computed Tomography

**Figure 2:**
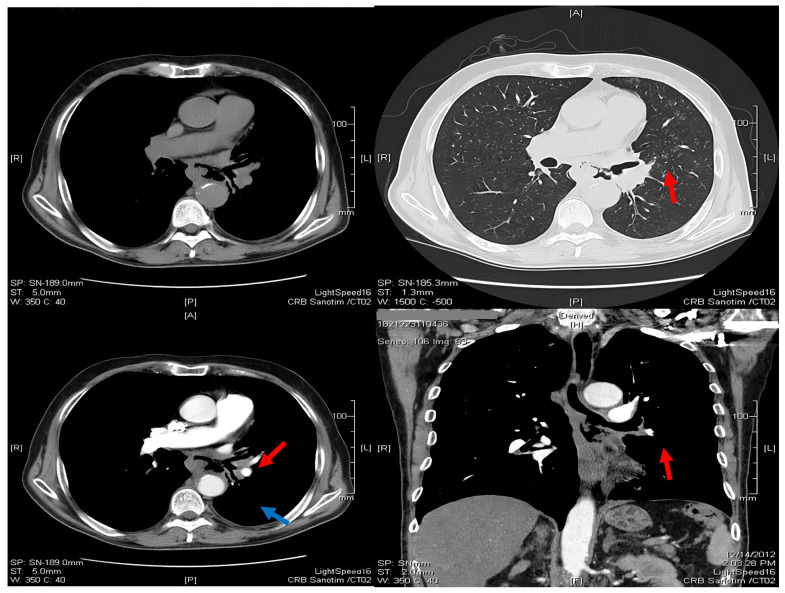
Axial and coronal CT sections showing the BEF between esophageal lumen and left main bronchus (red arrow) and the dense nodule (blue arrow).

A thoracic computed tomography (CT) scan revealed the presence of a mass in the lower left main bronchi and lobar inferior bronchi, which exhibited indistinct boundaries and an interior cavity with irregular walls. The mass appeared to coat and obstruct the bronchial outline in certain areas, extending to the esophageal wall. The esophagus was dilated and presented air leakage (Figure 2). The lungs showed a dense and irregularly shaped nodule measuring 3.5 by 2.6 cm, located in the posterior basal segment of the left inferior lobe, in contact with the parietal pleura, causing thickening in the infrahilar region (Figure 2).

### 2.2. Bronchoscopy

**Figure 3:**
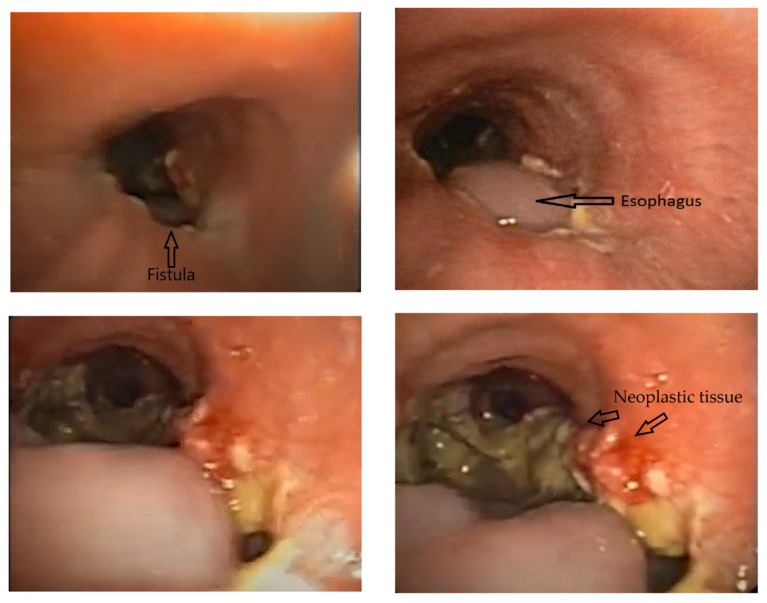
Bronchoscopic view showing the fistula in the bronchus and the neoplastic tissue.

Bronchoscopy revealed a giant broncho-mediastinal fistula that affected half of the distal inner wall of the primitive left bronchi, suggesting it was the result of pulmonary necrosis. The neoplastic tissue surrounded the fistula area and also extended to the left bronchi (Figure 3). During coughing, a relatively large segment of the esophagus prolapsed into the lumen of the left main bronchus due to the presence of the BEF (Figure 3). No additional intervention was carried out to address the tumor obstruction during bronchoscopy; instead, the case was referred to the gastroenterology department’s endoscopy unit for further assessment and management of the identified BEF.

Upper digestive endoscopy revealed an esophageal fistula with tumoral formation at 30 cm from the dental arch, with visualization of the bronchi; the fistula was approximately 3 cm in length (up to 33–35 cm from the incisors).

### 2.3. Bronchial Biopsy

**Figure 4:**
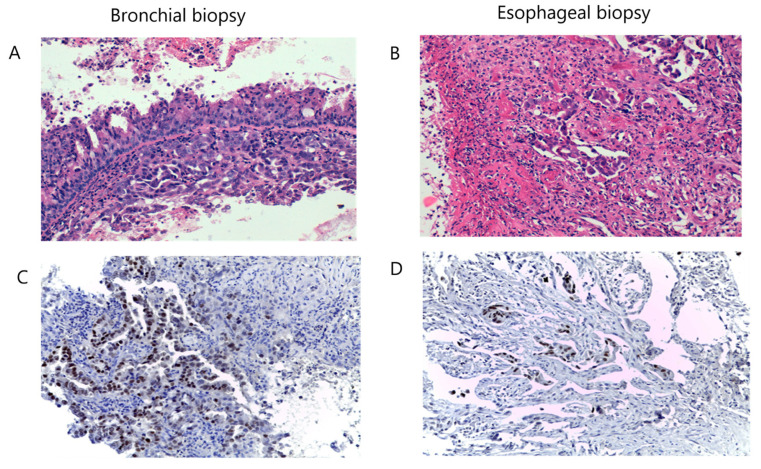
H&E staining of bronchial biopsied tissue (**A**), H&E staining of esophageal biopsied tissue (**B**), bronchial biopsy immune reaction with anti-TTF1 antibody (**C**) and esophageal biopsy-immune reaction with anti-TTF1 antibody (**D**). Magnification: ×20.

Figure 4 depicts the architecture and cellular morphology of bronchial biopsy versus esophageal biopsy. The histological aspect of the biopsied fragments suggested the presence of non-small cell lung carcinoma (NSCLC), identified as broncho-pulmonary adenocarcinoma according to hematoxylin and eosin (H&E) staining (Figure 4A) and immune reaction with anti-TTF1 antibody (Figure 4C). An esophagus biopsy revealed chorion invasion by the broncho-pulmonary adenocarcinoma (Figure 4B).

### 2.4. Treatment

**Figure 5:**
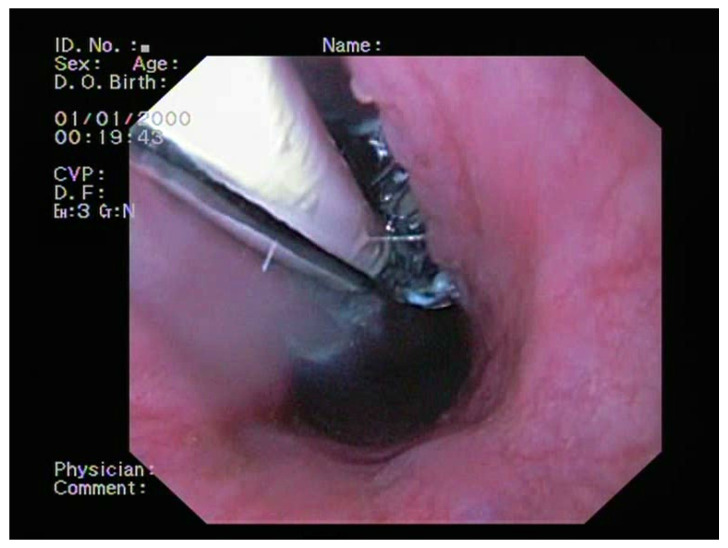
The endoscopic treatment of the BERF using implanting a partially covered Ultraflex stent.

Based on laboratory findings, clinical data, and imaging data, the endoscopic treatment of the BEF was selected using the partially covered Ultraflex Esophageal NG Stent System (Ultraflex, Boston Scientific, Marlborough, MA, USA) of size 12/9 cm and 28/23 mm in diameter, with proximal release and under direct endoscopic visualization (Figure 5). Ultraflex is a self-expanding metal stent (SEMS) meshed from a single strand of nitinol (5% nickel and 45% titanium) wire partially covered by a polyurethane sheath. The decision to prioritize the management of the BEF during bronchoscopy instead of simultaneously placing esophageal and tracheal stents was reached by considering all factors, including procedural risks, the patient’s clinical condition, and available resources. Consequently, this intervention was viewed as the most suitable and cautious approach.

**Figure 6:**
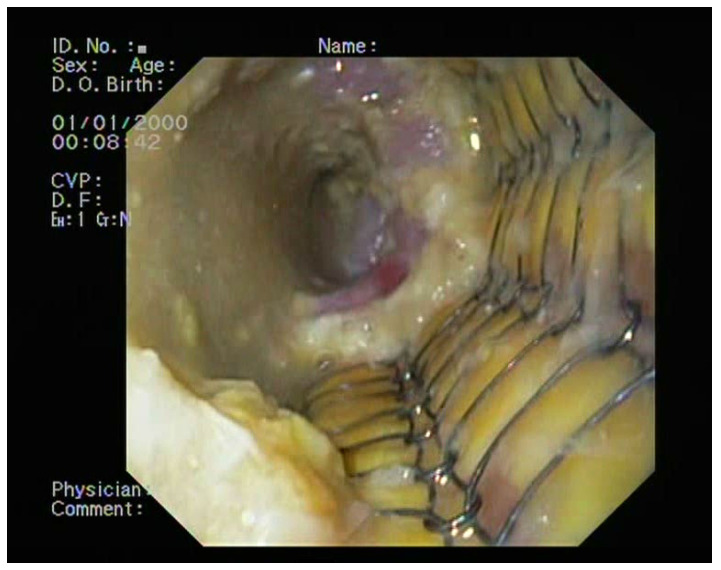
Endoscopic view of the stent implant at the 2-week follow-up.

The subsequent examination at 8 days revealed the presence of bezoars (food residues) that were easily removed using a polyp retrieval net (Figure 6). The post-procedure course of the condition was favorable, with significant improvement in terms of dysphagia, cough, and secondary suppuration. Oncological follow-up was recommended.

## 3. Discussion

This case report presents the complications of bronchopulmonary adenocarcinoma, a condition typically independent of interaction with the gastrointestinal tract. In this case, the complication was the formation of a BEF, which is a rare anatomical connection between the esophagus and bronchus. The available literature concerning BEF is notably limited compared to that on tracheoesophageal fistulas, particularly in the context of lung cancer, especially when the fistula involves the second- and third-degree bronchi, as observed in our case. This underscores the rarity of the presented case and highlights the need for further exploration in this area of study. Furthermore, the endoscopic approach to the esophagus distal to the fistula was challenging due to the presence of a flap produced by the fistula; the flap comprised the fistulized esophageal wall that obstructed the opening of the esophagus situated beneath the fistula.

Among all the esophageal respiratory fistulas, the most prevalent subtype is the trachea-esophageal fistula, which accounts for 52–57% of cases, followed by BEF, which accounts for 37–40% of cases [9]. The knowledge regarding esophageal respiratory fistulas primarily stems from the extended research on trachea–esophageal fistulas, while on BEF, the existing body of literature predominantly consists of sporadic case reports and limited observational studies due to their rare occurrence. Infections such as tuberculosis, syphilis, histoplasmosis, actino-mycosis, and candidiasis, although less common, are benign conditions that can cause BEF [10]. In areas where BEF is common, like India, tuberculosis should always be taken into account as a potential diagnosis. Additional instances of acquired cases of BEF encompass inflammatory disorders such as Crohn’s disease, Behect’s disease, Hodgkin’s disease, broncholithiasis, corrosive ingestion, and traumatic factors, including prolonged endotracheal intubation, blunt chest injury, and, although there is limited documentation in the literature, endoscopic intervention or esophageal foreign body [11,12,13,14,15].

The malignant esophago-respiratory fistulas arise more frequently due to malignancies affecting the esophagus compared to the fistulas produced by lung malignancies. In a study involving 207 malignant esophago-respiratory fistulas, Burt et al. reported that the esophagus was the primary tumor site in 77% of cases, whereas the lung was involved in only 16% of cases [16]. The available literature data are even scarcer in the case of malignant BEF due to lung cancer, thus emphasizing once more the rarity of the currently presented case; BEF rarely develops in lung cancer patients, and even more rarely in those with adenocarcinoma. Data obtained after analyzing a large group of patients with primary carcinoma revealed that the incidence of fistula development is 0.3%; of these patients, 58% had squamous cell carcinoma, 36% were diagnosed with adenocarcinoma, and the remaining 6% had oat cell carcinoma [16]. In individuals with malignancies, BEF can result from the direct invasion of the cancer or as a consequence of cancer treatment interventions, such as surgical procedures, chemotherapy, radiotherapy, and the use of esophageal stents. Sugimoto et al. reported the development of BEF in a male patient, aged 67, diagnosed with locally advanced squamous cell lung cancer, that was treated with steroids for radiation pneumonitis that occurred subsequently to severe radiotherapy [8]. BEF was rapidly diagnosed due to the presence of a cough that exacerbated following the consumption of liquids. This cough is observed in 56% of patients with BEF, while the presence of Ono’s sign (coughing while eating or drinking) is characteristic for these individuals [8]. Similarly, in a patient with pulmonary adenocarcinoma, BEF was formed after the first course of treatment with platinum-based chemotherapy containing bevacizumab; however, despite the concurrent tracheobronchial and esophageal stenting procedures that resulted in a notable improvement in the patient’s condition, the patient died due to massive hemoptysis [17]. In a small cell lung carcinoma patient’s case, concurrent treatment with radiotherapy and chemotherapy led to the formation of BEF, which was diagnosed upon the patient’s arrival at the hospital, presenting with symptoms of cough and dysphagia [18].

Complications associated with BEF encompass a spectrum of symptoms, including dysphagia, productive cough, cough upon drinking fluids, shortness of breath, purulent sputum, generalized weakness, anorexia, weight loss that can lead to cachexia/emaciation, bronchopulmonary infections, asphyxiation through food aspiration into the trachea, aspiration pneumonia, anemia due to chronic blood loss, and gastric distension [4,19,20]. In our particular case, the fistula was discovered after a long period during which the patient experienced respiratory distress, predominantly characterized by coughing and recurrent infections, alongside dysphagia and episodes of choking. Prompt diagnosis and treatment are essential to prevent complications and enhance the quality of life for these patients [21].

Regarding therapeutic possibilities, in the absence of a standardized protocol, there are various conservative techniques available, including stent implantation, fistula sealing using adhesive agents, application of different types of clipping devices (such as ordinary clips or the Ovesco clipping device), or surgical intervention through excision of the fistula. The management of BEF depends on the extent of symptomatology, the specific anatomical site of the fistula, and the overall health status of the individual. As reported by Khuwaja et al., even if BEF symptoms are present and affect the patient’s quality of life, some of these patients are not good candidates for the surgical repair of BEF [19]. In a similar scenario to the one described in our study, the occurrence of BEF resulted in the necrosis and perforation of the bronchial wall, as well as the prolapse of the esophageal wall into the bronchial lumen. Due to the considerable size of the fistula, conservative treatments were deemed unfeasible, leading to the referral of patients for surgical intervention [6]. In our case, we chose endoscopic stent insertion as a palliative and conservative intervention due to its minimal invasiveness.

Different manufacturers all over the world produce numerous stent variations. The available stents exhibit variations in terms of stent material, design, luminal diameter, radial force applied, flexibility, and extent of shortening following usage. Stent insertion is associated with a significant improvement in patients′ BEF symptoms even in the case of late-stage cancer, as described in a 65-year-old male patient with stage IIIB squamous cell carcinoma [20].

Based on their composition, there are two primary categories of stents: plastic stents and metal stents. Self-expanding plastic stents (SEPSs) consist of a polyester braid that is fully enveloped in a silicone membrane. The device’s proximal end is flared for secure placement and consistent closure, with radio-opaque markers situated at both ends and in the center to facilitate accurate positioning [22]. Despite these features, the main disadvantage of SEPSs is their tendency to migrate, with reported rates between 5 and 23% [23]. Even though the entire silicone covering facilitates easy retrieval, it also increases the likelihood of migration due to the minimum granulation reaction [22]. In addition, SEPS occlusion was reported in 12.1% of cases due to tumor overgrowth, and the general SEPS reintervention rate was 21.1% [24]. Concurrently, self-expanding metal stents (SEMSs) are available in three different forms: totally covered, partially covered, and uncovered. There is empirical evidence indicating that the performance of covered stents surpasses that of uncovered ones [25,26,27]. Partially covered stents are superior in the palliation of malignant dysphagia, relieving the swallowing difficulties caused by unresectable obstructive esophageal cancers. The occurrence of recurrent dysphagia due to restenosis was substantially more frequent in patients with uncovered stents compared to those with partially covered stents (37% versus 8%) [26]. Moreover, as reported by Vakil et al., the occurrence of tumor ingrowth was considerably higher in the group of patients with uncovered stents (9 out of 30) compared to the group with covered stents (1 out of 32) [25]. The rate of reinterventions for tumor ingrowth was considerably higher in the group of patients who had uncovered stents (27%) compared to the group with covered stents (0%) [25]. A similar incidence of tumor ingrowth was reported by Adam et al., where 26% of the patients with uncovered stents developed tumor ingrowth [28]. Furthermore, an elevated risk of major complications was observed in the patients receiving SEPS compared to those who received SEMS [29]. When comparing the partially covered SEMS with the fully covered SEMS, a recent meta-analysis revealed that there were no differences between the patients from the two groups in terms of dysphagia improvement, stent occlusion, and migration [30].

The potential problems associated with SEMS include immediate issues, such as aspiration, malposition, delivery system entrapment, stent dislodgement, and perforation [31]. Bleeding, chest pain, and nausea can occur within the first week after insertion, while recurrent dysphagia, migration, bleeding, and gastroesophageal reflux disease can occur as delayed complications [32]. Patel et al. reported the formation of BEF as a delayed complication of a fully covered SEMS insertion. The patient, diagnosed with non-small cell lung carcinoma, had a SEMS placed in the proximal region of her esophagus to alleviate the esophageal dysphagia that occurred as a result of radiation therapy [33]. After 1 year post insertion, the fully covered SEMS migrated distally and caused the formation of her BEF; the fully covered SEMS was replaced with a partially covered SEMS that led to the resolution of the fistulous tract [33]. Even though the early complications after SEMS insertion are rare, delayed complications have been reported in up to 63% of cases [34]. Dysphagia leads to anorexia, and anorexia to cachexia; it is known that in cancer cachexia, inflammatory cytokines decrease cellular catabolism, resulting in weight loss [35]. In our case, the patient, unable to orally ingest food, received parenteral nutrition as it seemed the most feasible option due to concerns surrounding enteral nutrition’s higher risks, including reflux, aspiration, reduced tolerability, and potential tube and pump issues. Moreover, percutaneous gastrostomy/jejunostomy carried the risk of complications related to stoma implantation. Although parenteral nutrition has some disadvantages, such as its high cost, risk of thrombophlebitis, and lower efficacy, it remains the preferred choice. The literature data present various cases regarding the management of stent-related tracheoesophageal fistula in terms of patients feeding and enteric nutrition [36,37,38]; however, limited data are available in the case of BEF. As reported by Patel et. al., in the case of a patient with non-small cell lung carcinoma, the fully covered SEMS placed to improve esophageal dysphagia migrated distally and led to the formation of a BEF [33]. In this case, the fully covered stent was replaced with a partially covered one, while a gastrostomy/jejunostomy tube was placed to facilitate feeding [33].

For our patient, we chose to place a partially covered esophageal stent as it is a less invasive procedure. The margins of a partially covered stent are implanted directly into the normal esophageal mucosa, preventing stent migration, thus ensuring that the patient will be able to eat without the risk of choking. The stent we used offers various advantages, such as obstruction elimination, an increased degree of palliation with significant improvement in dysphagia, tumor ingrowth prevention, a low migration rate, and a lower complication rate. Another advantage of the partially covered stent was that it sealed the BEF with the covered part, thus improving both respiratory and digestive symptoms. Usually, prosthesis implantation involves the placement of prostheses with distal deployment under a radiological screen. In this instance, we present the implant procedure of a partially covered prosthesis, with proximal deployment, using direct endoscopic visualization without the use of a radiological screen. This procedure offers a cost-effective and radiation-free alternative to the classic technique that involves C-arm radiology. Furthermore, this approach for patients with BEF is highly beneficial and can be employed especially in healthcare facilities lacking radiology services.

## 4. Conclusions

This case report presents the therapeutic approach employed in a patient presenting with a BEF secondary to bronchopulmonary adenocarcinoma that involved second- and third-degree bronchi, where palliative intervention stands as the sole viable treatment option. In this rare situation, stent implantation emerged as the preferred therapeutic modality, representing, in our view, the only therapeutic possibility for the correction of dysphagia and respiratory dysfunction. Unlike traditional methods, we used direct endoscopic visualization for proximal deployment, eliminating the need for radiation exposure and offering cost-effective advantages, particularly for facilities without radiology services. Choosing the appropriate stent and its meticulous implantation yielded substantive enhancements in the patient’s quality of life and protracted survival, and it also facilitated the continuation of other palliative treatment modalities.

## Figures and Tables

**Table 1:** Blood test results.

	Values
Erythrocytes	3,940,000/mm^3^
Hemoglobin	14.5 g/dL
Hematocrit	42%
Leukocytes	5730/mm^3^
Thrombocytes	304,000/mm^3^
VSH	9 mm/h

## Data Availability

The data presented in this study are available on request from the corresponding author. The data are not publicly available due to privacy.
